# Monthly Summary

**Published:** 1878-11

**Authors:** 


### MONTHLY SUMMARY.

*Chloroform.—Rules for Administration.—Its Peculiarties Nationally.*—Dr. M. F. Comes, (*American Med. Bi- Weekly,*) thinks that the dangers from chloroform arise from an accumulation of it in the system, and that rapid administration of it is safest. Resistance to its inhalation may be overcome by pressure upwards and backwards on the ensiform cartillage. Irregularity in the respiration or circulation is more easily remedied if the system is not charged with the drug, as it is apt to be when administered slowly. Little attention need be paid to diseased conditions of the heart or lungs, but the pulse should be carefully watched; and it is a point of importance to have some previous knowledge of this indication as the part of the subject, as irregularity or intermittency of pulse is not unfrequent in persons enjoying good health, but marked irregularity while the anæsthetic is being administered is suspicious, and significant of danger. Sudden increase in frequency and corresponding diminution in volume is likewise alarming, but is a rare symptom. The face is a good index of the condition, and pallor, is suggestive of interference with a normal condition of affairs. [Pallor is indicative sometimes of nausea—see Chisholm on Chloroform. Eds.]

Dr. Comes thinks nationality has much to do with the susceptibility of individuals to anæsthetics, and classes the Irish as most difficult to subject to their influence; and in this respect they rank with Americans, who are strongly resistant also. Germans yield easily to chloroform, and the Negro most easily of all.

Young persons are much more susceptible to its influence than adults; extreme old age does not lessen this resistance, but debility does, and delicate persons yield more readily than robust constitutions.

[We add that nervous and excitable cases are difficult to manage, being brought under the influence of anæsthetics slowly, and with great resistance. The readiness with which children yield to chloroform is quite remarkable, and the promptness with which they rally is equally so. Knowledge of these facts should influence us in cases of painful extraction, when the little sufferer suffers equally from fear of the operation and operator. The administration of chloroform to a small child may be said to be almost absolutely devoid of danger.]

*A Peculiar Mouth Affection.*—Dr. Geo. Y. Hunter, of Bengal, describes a peculiar mouth affection which attacked the troops in India, which was characterized by slight elevation of fungiform and filliform papillæ, followed by stripping or peeling off of patches of epithelium chiefly in the middle of the dorsum of the tongue, with fissures in the direction of the muscular fibres; the whole mouth is sore, and the gums are blueish and ulcerated at the junction of the teeth; no pain is present, nor does the thermometer show general elevation of temperature, though there is a slight increase of heat locally. The pharynx and throat become involved as the disease progresses, with hoarseness and swelling in the sub-maxillary region. The disease reached its maximum of intensity in about a fortnight. The cause is attributed to a change to unaccustomed food.

---

*Nitrite of Amyl and Chloroform as an Anæsthetic.*—L. B. Balliet, M. D., in the *Med. and Surg. Reporter,* says:

In my practice of over twenty-four years I have, until recently, used chloroform as an anæsthetic in all surgical cases and convulsions of children. The past six months, with the object of lessening the dangers of asphyxia by this powerful anæsthetic, I added to the ounce of chloroform sixteen drops of nitrite of amyl. The result thus far is apparently most satisfactory. Nevertheless, further careful tests are needed to fully confirm my views of this combination as a safe anæsthetic. I therefore ask surgeons to give this a fair trial, and report their experiences in regard to its action on the heart, respiration and circulation, and compare it carefully with the symptoms produced when chloroform alone is used. I shall still continue the use of this compound anæsthetic, but may vary the proportions of the nitrite of amyl in particular cases.

---

*The Bael as an Astringent.*—This Indian drug is rising in popularity in England. The tree of which the bael is the fruit grows to a large size, and is sacred to Siva. The fruit, the size of an orange, contains an aromatic essential oil, with pungent and astringent substances. It is employed especially as a carminative, as an astringent, and an aperient in constipation, being given especially in dysentery, diarrhœa, and indigestion; in the latter to regulate the bowels. In the later stage of dysentery, when diarrhœa, simple or dysenteric, continues with exhaustion, Sir Joseph Fayrer especially recommends it. It has been occasionally given in this country, and a formula exists in the British Pharmacopœia for the preparation of a liquid extract, but the value of this preparation is regarded as somewhat doubtful. In India it is frequently given in the form of sherbet or marmalade. —*Med. and Surg. Reporter.*

